# Cellular senescence induced by S100A9 in mesenchymal stromal cells through NLRP3 inflammasome activation

**DOI:** 10.18632/aging.102409

**Published:** 2019-11-14

**Authors:** Lei Shi, Youshan Zhao, Chengming Fei, Juan Guo, Yan Jia, Dong Wu, Lingyun Wu, Chunkang Chang

**Affiliations:** 1 Department of Hematology, Shanghai Jiao Tong University Affiliated Sixth People’s Hospital, Shanghai 200233, China

**Keywords:** myelodysplastic syndromes, mesenchymal stromal cells, S100A9, cellular senescence, NLRP3

## Abstract

Bone marrow stromal cells from patients with myelodysplastic syndrome (MDS) display a senescence phenotype, but the underlying mechanism has not been elucidated. Pro-inflammatory signaling within the malignant clone and the bone marrow microenvironment has been identified as a key pathogenetic driver of MDS. Our study revealed that S100A9 is highly-expressed in lower-risk MDS. Moreover, normal primary mesenchymal stromal cells (MSCs) and the human stromal cell line HS-27a co-cultured with lower-risk MDS bone marrow mononuclear cells acquired a senescence phenotype. Exogenous supplemented S100A9 also induced cellular senescence in MSCs and HS-27a cells. Importantly, Toll-like receptor 4 (TLR4) inhibition or knockdown attenuated the cellular senescence induced by S100A9. Furthermore, we showed that S100A9 induces NLRP3 inflammasome formation, and IL-1β secretion; findings in samples from MDS patients further confirmed these thoughts. Moreover, ROS and IL-1β inhibition suppressed the cellular senescence induced by S100A9, whereas NLRP3 overexpression and exogenous IL-1β supplementation induces cellular senescence. Our study demonstrated that S100A9 promotes cellular senescence of bone marrow stromal cells via TLR4, NLRP3 inflammasome formation, and IL-1β secretion for its effects. Our findings deepen the understanding of the molecular mechanisms involved in MDS reprogramming of MSCs and indicated the essential role of S100A9 in tumor-environment interactions in bone marrow.

## INTRODUCTION

Myelodysplastic syndromes (MDS) are a diverse group of clonal hematopoietic malignancies characterized by ineffective hematopoiesis, progressive bone marrow failure, cytogenetic, molecular abnormalities, and variable risk of progression to acute myeloid leukemia.

The bone marrow microenvironment plays a key role in the pathogenesis of MDS. MSCs are major cellular components of the bone marrow microenvironment, and MSCs isolated from patients with MDS (MDS-MSC) display a distinct phenotype and growth characteristics and singular genomic changes. We have previously demonstrated that MDS-MSCs have compromised colony-forming and proliferation ability in vitro, and display a senescence phenotype with activated p53-p21 senescent signal being especially prominent in lower-risk MDS-MSCs [1].

The pathogenesis of MDS has been associated with an aberrant cross talk between hematopoietic elements and the stromal compartments. Moreover, patient-derived hematopoietic cells could instruct healthy MSCs to acquire MDS-MSC like features [2]. Leukemia cells promote the progression of leukemia and inhibit normal hematopoietic function by “reprogramming” bone marrow microenvironment, including MSCs, for instance, leukemia cells regulate bone marrow microenvironment by secreting chemokine (C-C) ligand 3 (CCL3) and stem cell factors (SCF), thus inhibiting normal bone marrow hematopoietic function [3]; and change the expression of important adhesion chemokine, inhibit normal hematopoiesis, and support leukemia generation [4]; and promote the formation of osteoblasts with abnormal MSC function through thrombopoietin (TPO)/CCL3 or direct adhesion. The abnormal function of osteoblasts is manifested by abnormal signaling of transforming growth factor β (TGF-β), NOTCH, and inflammatory pathways, resulting in impaired normal hematopoietic function, leukemic stem cell support, and promotion of bone marrow fibrosis development [5]. In chronic myeloid leukemia, leukemia cells reduce the expression of stromal cell derived factor 1 (SDF-1) in stromal cells by secreting granulocyte colony-stimulating factor (G-CSF), thus affecting the homing and stabilization of hematopoietic cells [6].

More importantly, Medyouf et al. found that MDS tumor cells and the MDS cell line MDS-L can “reprogram” normal MSCs to obtain MDS-MSC-like characteristics, such as high leukemia inhibitory factor (LIF) expression levels, suggesting that MDS-MSC senescence may be related to MDS clonal cells [2]. Tumor cells in many solid tumor types have been shown to induce senescence of peripheral fibroblasts and further affect disease prognosis. Increased MSC senescence is a common phenomenon in MDS, suggesting that it may be caused by a common signal of MDS clonal cells.

Pro-inflammatory signaling within the malignant clone and the bone marrow microenvironment has been identified as a key pathogenic driver of MDS [7]. S100A9, an important member of the calcium binding protein S100 family, is secreted mainly by inflammatory, tumor, and stromal cells. The natural state of the protein is dependent on the environment it resides. It can bind to TLR4 in a paracrine or autocrine manner to activate NLRP3 inflammasomes. Inflammasomes are multiprotein oligomer complexes and important components of the innate immunity network that are triggered during “sterile” inflammation in response to damage associated molecular patterns (DAMPs) [8–10]. The best characterized NLR, NLRP3, is a redox-sensitive cytosolic sensor that recruits the apoptosis-associated speck-like protein containing a CARD (ASC) adaptor protein. NLRP3 is activated by diverse DAMP signals, including S100A9 homodimers and S100A8/9 heterodimers that function as alarmins to generate reactive oxygen species (ROS) [11].

Our study aimed to explore the role of S100A9 in MSC senescence and to identify its potential mechanisms.

## RESULTS

### S100A9 induces cellular senescence in bone marrow stromal cells

S100A9 secreted from tumor cells is an important regulator of tumor microenvironment [12]. To identify the potential senescence-inducing effect of MDS-derived S100A9 on MSCs, we conducted experiments with the human stromal cell line HS-27a and primary normal human MSCs.

**Figure 1:**
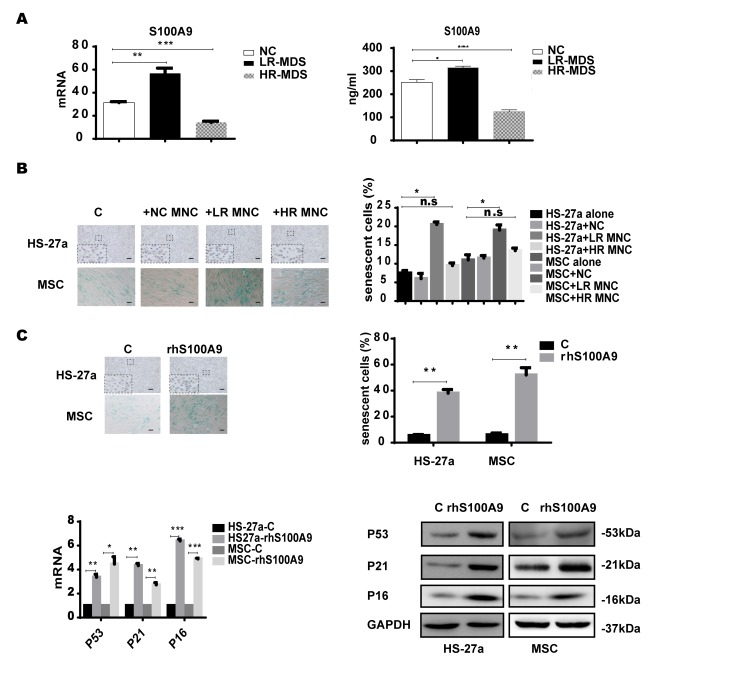
**S100A9 induces cellular senescence in bone marrow stromal cells.** (**A**) S100A9 expression in BM-MNCs isolated from MDS patient specimens (n = 19 LR-MDS, and n = 13 HR-MDS) compared with the expression in normal control BM-MNCs (n =20) as determined with qPCR (left). S100A9 levels in bone marrow supernatant from MDS patient specimens (n = 16 LR-MDS, and n = 8 HR-MDS) compared with the levels in normal BM-MNCs (n =10) as measured with ELISA (right). (**B**) HS-27a cells and MSCs were co-cultured with normal control BM-MNCs and MDS BM-MNCs. Senescent cells (green) were tested after 72 h. The number of senescent cells from at least 500 cells in 10 randomly chosen fields was used to calculate their percentage (40 × magnification). (**C**) Cells were treated with rhS100A9 (200 ng/ml for HS-27a, 500 ng/ml for MSC) for 72h. Subsequently, senescent cells were counted and presented in graphs (40 × magnification). Finally, p53, p21, and p16 levels were measured using qPCR and western blot. Data are expressed as mean ± SD from three experiments. *P<0.05, **P<0.01, and ***P<0.001.

Firstly, we measured the S100A9 expression levels in MDS-BM-MNCs with qPCR and found that S100A9 were highly-expressed in lower-risk MDS-BM-MNCs, but showed no difference between higher-risk MDS-BM-MNCs and normal controls; similar results were obtained with ELISA (Figure 1A). Next, we used a transwell membrane co-culture system, in which MDS-BM-MNCs and MSCs were cultured on the insert membrane and in the lower chamber, respectively, the membrane allows molecular <0.4um to transport.

SA-β-gal staining assay was used to determine cellular senescence in MSCs. SA-β-gal, shorted for senescence-associated β-galactosidase, is a product of lysosome and expressed by senescent, but not presenescent, quiescent, terminally differentiated cells, the presence of the SA-β-gal biomarker is independent of DNA synthesis and generally distinguishes senescent cells from quiescent cells [13]. Today, SA-β-gal is the most common and most useful marker to screen out senescent cells [14–15] In our study, senescent MSCs (SA-β-gal positive cells) were increased when co-cultured for 72h with lower-risk MDS-BM-MNCs but not with higher-risk MDS-BM-MNCs and normal control BM-MNCs (Figure 1B).

To further ascertain the role of S100A9 in MSC senescence, 200 ng/ml and 500 ng/ml rhS100A9 were administered to HS-27a and primary MSC cells, respectively. The proportion of senescent cells was significantly increased after rhS100A9 treatment for 72h. It has been demonstrated that p53, p21, and P16 have a direct interaction with senescence regulation processes. We also detected increased p53, p21, and p16 mRNA and protein levels using qPCR and western blot, respectively The levels of p53, p21 and p16 were significantly increased when treated with rhS100A9 (Figure 1C).

### TLR4 is required for S100A9-induced cellular senescence

**Figure 2:**
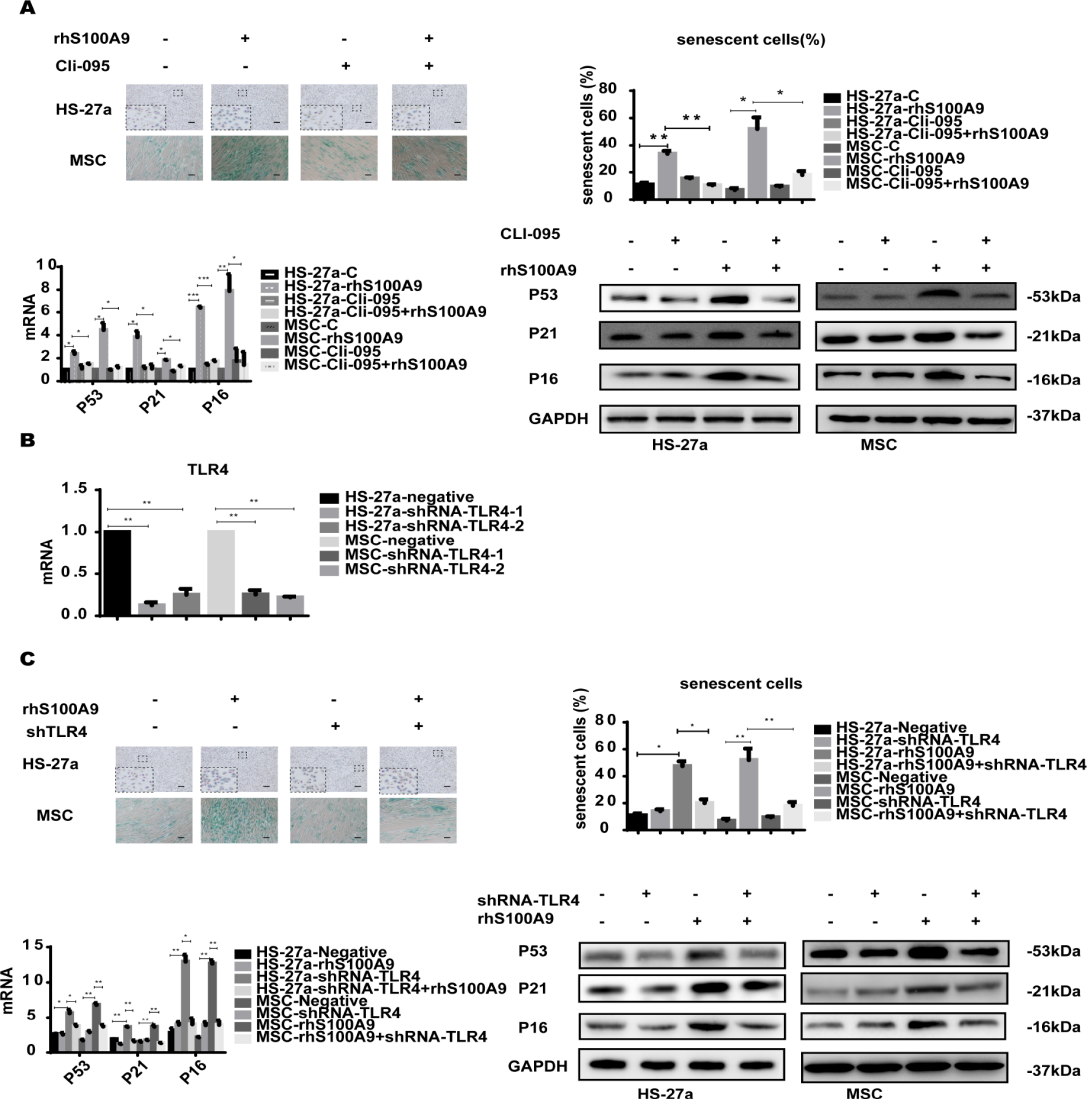
**TLR4 is required for S100A9-induced cellular senescence.** (**A**) Cells were pretreated with CLI-095 (1 μg/ml) for 2 h and then treated with rhS100A9 for 72h. Senescent cells were counted and presented in graphs (40 × magnification). The expression of p53, p21, and p16 was measured using qPCR and western blot. (**B**) The RNAi efficiency of TLR4 was assayed utilizing qPCR. (**C**) TLR4 was knocked down, and cells were treated with rhS100A9 for 72h. Senescent cells were counted and presented in graphs (40 × magnification). Next, p53, p21, and p16 levels were measured utilizing qPCR and western blot. Data are expressed as mean ± SD from three experiments. *P<0.05, **P<0.01, and ***P<0.001.

S100A9 is an endogenous activator of TLR4 [16], a receptor also expressed in MSCs [17]. To determine whether TLR4 is responsible for the cellular senescence induced by S100A9, the TLR4 inhibitor CLI-095 and shRNA-TLR4 were used in this assay. Cells were pretreated with 1 μg/ml CLI-095 for 2 h, then with rhS100A9. As expected, after 72h, the S100A9-induced cellular senescence was attenuated as evidenced by the decreased proportion of senescent cells and reduced p53, p21, and p16 levels (Figure 2A). Meanwhile, we constructed two retrovirus-based RNAi vectors that transfect cells with high efficiency, cells were transfected with the retroviral supernatant containing shRNA specific to the human TLR4. TLR4 was reduced on average by approximately 80% as evaluated by qPCR (Figure 2B). Similar to previously obtained results, TLR4 knockdown decreased the proportion of senescent cells and p53, p21, and p16 levels induced by S100A9 (Figure 2C).

### NLRP3 inflammasome and IL-1β secretion is involved in S100A9-induced cellular senescence

**Figure 3:**
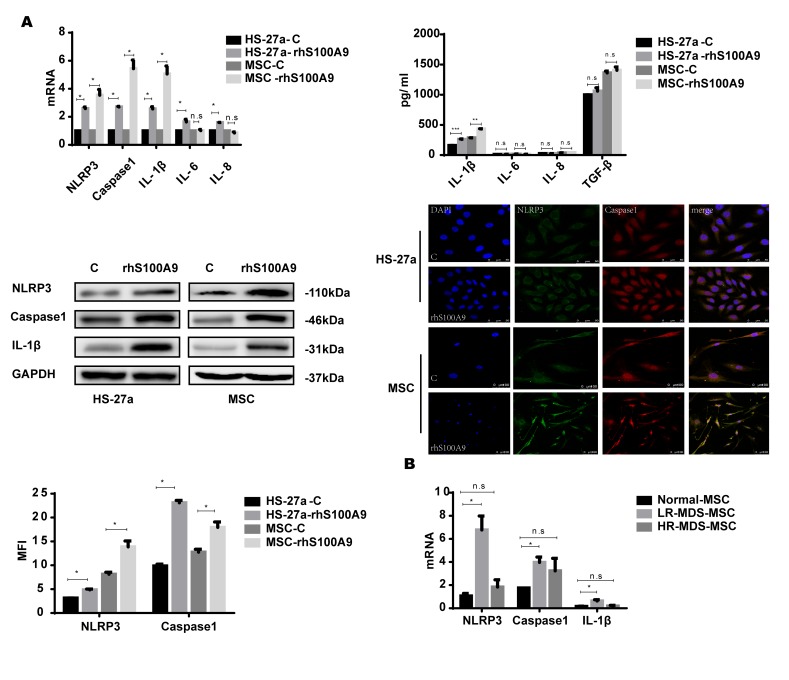
**NLRP3 inflammasome and IL-1β secretion is involved in S100A9-induced cellular senescence.** (**A**) Cells were treated with rhS100A9 for 72h. NLRP3, Caspase-1, IL-1β levels were measured using qPCR and western blot. IL-1β, IL-6, IL-8, and TGF-β expression and secretion was measured utilizing qPCR and ELISA. Representative confocal fluorescence micrograph of Caspase-1 and NLRP3 expression in cells treated with S100A9 (DAPI, blue; Caspase-1, red; and NLRP3, green; merged images show inflammasome formation; scale bars: HS-27a 50 μm and MSC 100 μm). (**B**) qPCR analyses of NLRP3, Caspase-1, and IL-1β expression in BM-MSCs isolated from MDS patient specimens (n = 18 LR-MDS, and n = 12 HR-MDS) compared with the levels in normal control BM-MNCs (n =18). Data are expressed as mean ± SD from three experiments. *P<0.05, **P<0.01, and ***P<0.001.

NLRP3 inflammasomes are associated with cellular senescence and inflammatory diseases [18–20], and NLRP3 is activated by diverse DAMP signals including S100A9 homodimers [7, 21–23]. In addition, the NLRP3 inflammasome controls inflammatory responses and activates Caspase-1 for the subsequent maturation of pro-inflammatory cytokines, for instance, IL-1β [24–25]. Thus, S100A9 may induce NLRP3 inflammasome formation and possibly also pro-inflammatory cytokine secretion in the cellular senescence process. In our study, the levels of NLRP3, Caspase-1, and IL-1β were markedly increased by S100A9. However, IL-6 and IL-8 were upregulated in HS-27a cells but remained unchanged in primary MSCs. We further examined the secretion of the pro-inflammatory cytokines by ELISA; IL-1β secretion was upregulated, while the levels of IL-6, IL-8, and TGF-β remained unchanged. Moreover, confocal fluorescence microscopy confirmed that S100A9 treatment promotes NLRP3 inflammasome formation, and further proved by MFI (Median Fluorescence Intensity) analysis (Figure 3A).

Further, we detected the expression of NLRP3, Caspase-1, and IL-1β in MDS-MSCs with qPCR. Consistent with our previous results, NLRP3, Caspase-1, and IL-1β levels were higher in lower-risk MDS-MSCs but showed no differences between higher-risk MDS-MSCs and normal controls (Figure 3B).

### S100A9-induced cellular senescence, NLRP3 inflammasome formation, and IL-1β secretion require ROS

ROS, a group of highly reactive chemicals containing oxygen, are produced both endogenously and exogenously. Increased ROS levels have repeatedly been observed under iron overload conditions, which is a common phenomenon in MDS patients [26]. Furthermore, highly-expressed ROS has been detected in MDS patients as well [27]. ROS production has recently been identified as a downstream target of the S100A9/ CD33 pathway [28], and NLRP3 inflammasome formation usually requires a second signal delivered from ROS production [29–31]. In addition, ROS has been confirmed as another important factor, which can mediate NLRP3 inflammasome formation [9, 32].

**Figure 4:**
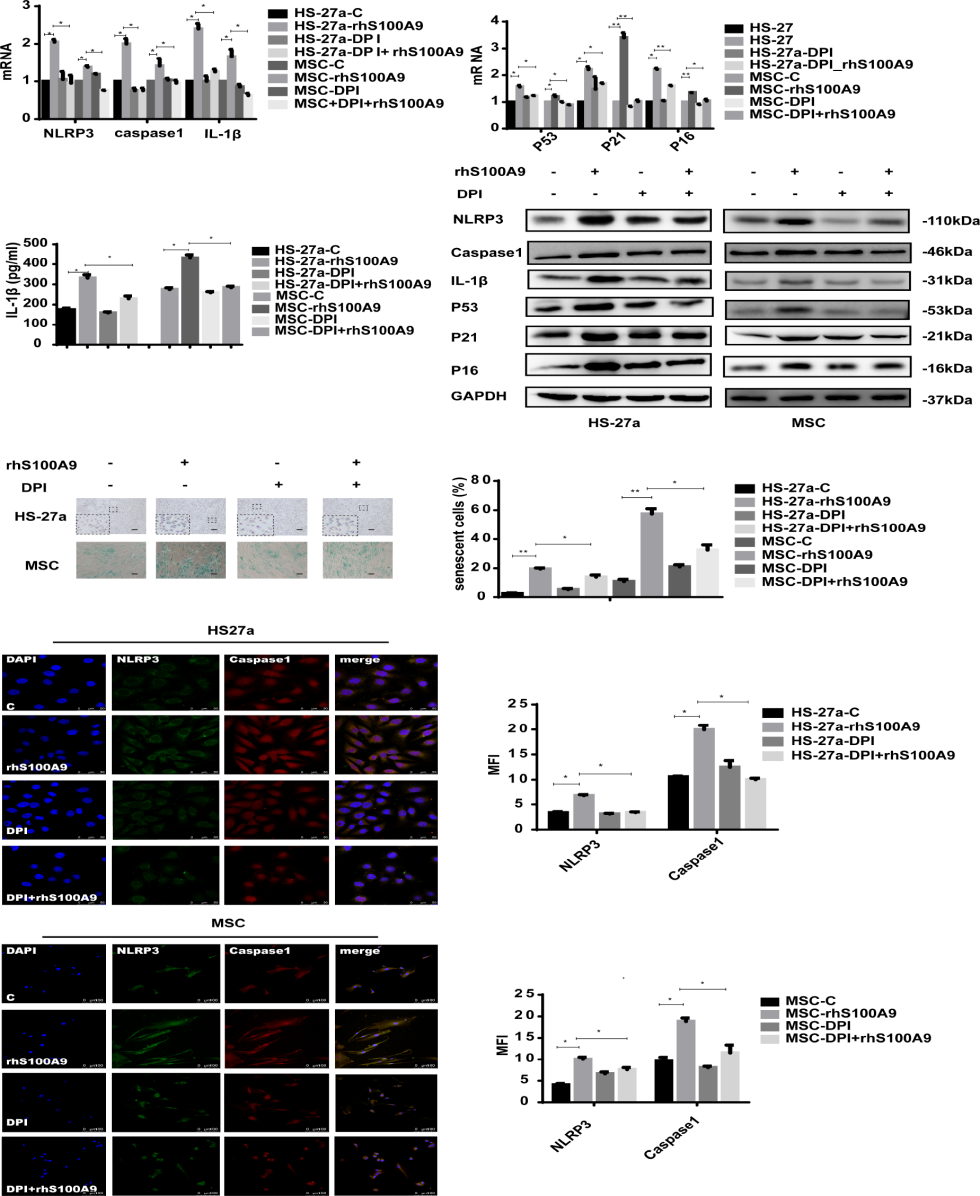
**S100A9-induced cellular senescence, NLRP3 inflammasome formation, and IL-1β secretion require ROS.** Cells were pretreated with DPI for 2 h and then treated with S100A9 for 72h. NLRP3, Caspase-1, IL-1β, p53, p21, and p16 expression was tested using qPCR and western blot. IL-1β secretion was measured utilizing ELISA. Senescent cells were counted and presented in graphs (40×magnification). Representative confocal fluorescence micrograph of Caspase-1 and NLRP3 expression (scale bars: HS-27a 50 μm and MSC 100 μm). Data are expressed as mean ± SD from three experiments. *P<0.05, **P<0.01, and ***P<0.001.

In our study, the mitochondrial ROS levels was increased and anti-oxidative enzyme superoxide dismutase (SOD), glutathione (GSH), and catalase (CAT) levels was reduced when treated with S100A9 (Supplementary Figure 1). To further clarify the role of ROS in S100A9-induced NLRP3 inflammasome formation, ROS was inhibited by DPI (10 μM/ml). As indicated in Figure 4, DPI abrogated NLRP3, Caspase-1, and IL-1β expression induced by S100A9. IL-1β secretion and NLRP3 inflammasome formation were also reduced.

Furthermore, we detected the influence of DPI treatment on MSC cellular senescence. As expected, DPI decreased the proportion of senescent cells and p53, p21, p16 expression levels induced by S100A9.

### NLRP3 overexpression induces IL-1β secretion and cellular senescence

**Figure 5:**
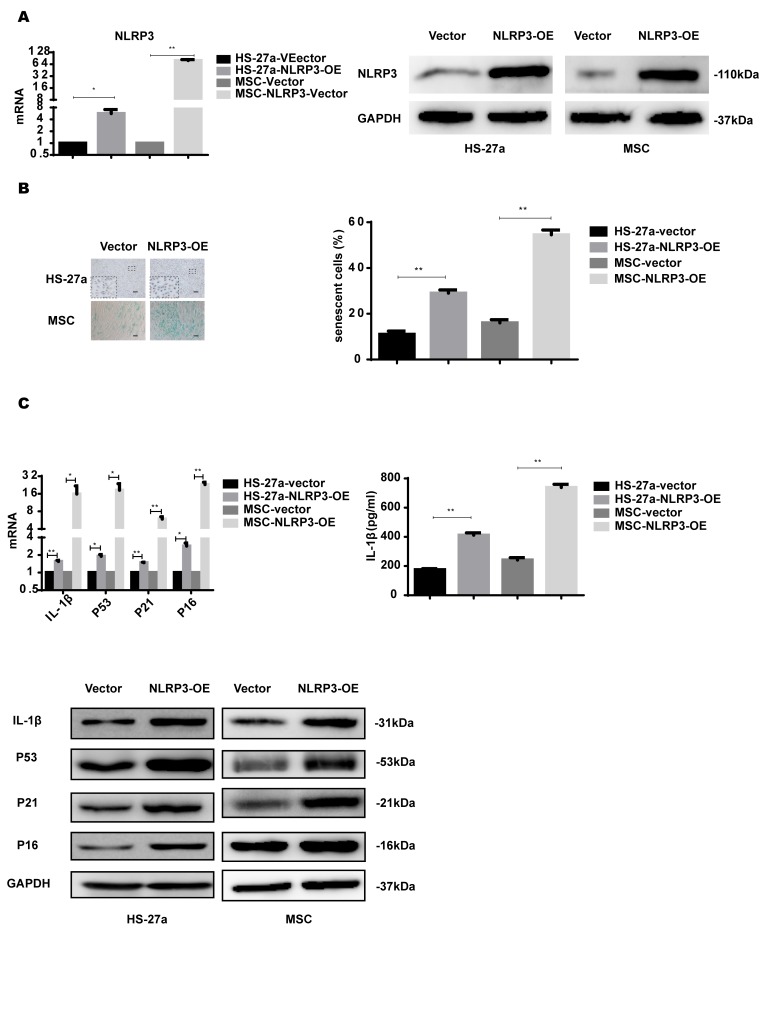
**NLRP3 overexpression induces IL-1β secretion and cellular senescence.** (**A**) The efficiency of NLRP3 overexpression was assayed with qPCR and western blot. (**B**) Senescent cells were counted and visualized in graphs (40 × magnification). The levels of p53, p21, p16, and IL-1β were measured by qPCR and western blot. Data are expressed as mean ± SD from three experiments. *P<0.05, **P<0.01, and ***P<0.001.

NLRP3 inflammasome formation leads to IL-1β production and is important for inflammasome priming and assembling. To further confirm the role of NLRP3 in S100A9-induced cellular senescence, HS-27a and primary MSC cells were transfected with retrovirus carrying the NLRP3 gene. NLRP3 expression was considerably increased at both mRNA and protein levels (Figure 5A).

NLRP3 overexpression increased IL-1β expression levels and secretion. The proportion of senescent cells was higher in the NLRP3-OE group than the vector group. Furthermore, p53, p21, and p16 levels were increased as well, as shown in Figure 5B.

### IL-1β is sufficient to induce cellular senescence

IL-1β is an important stromal growth factor for the maintenance of multipotent mesenchymal stromal cells and enhances their ability to sustain HSCs [33, 34].

**Figure 6:**
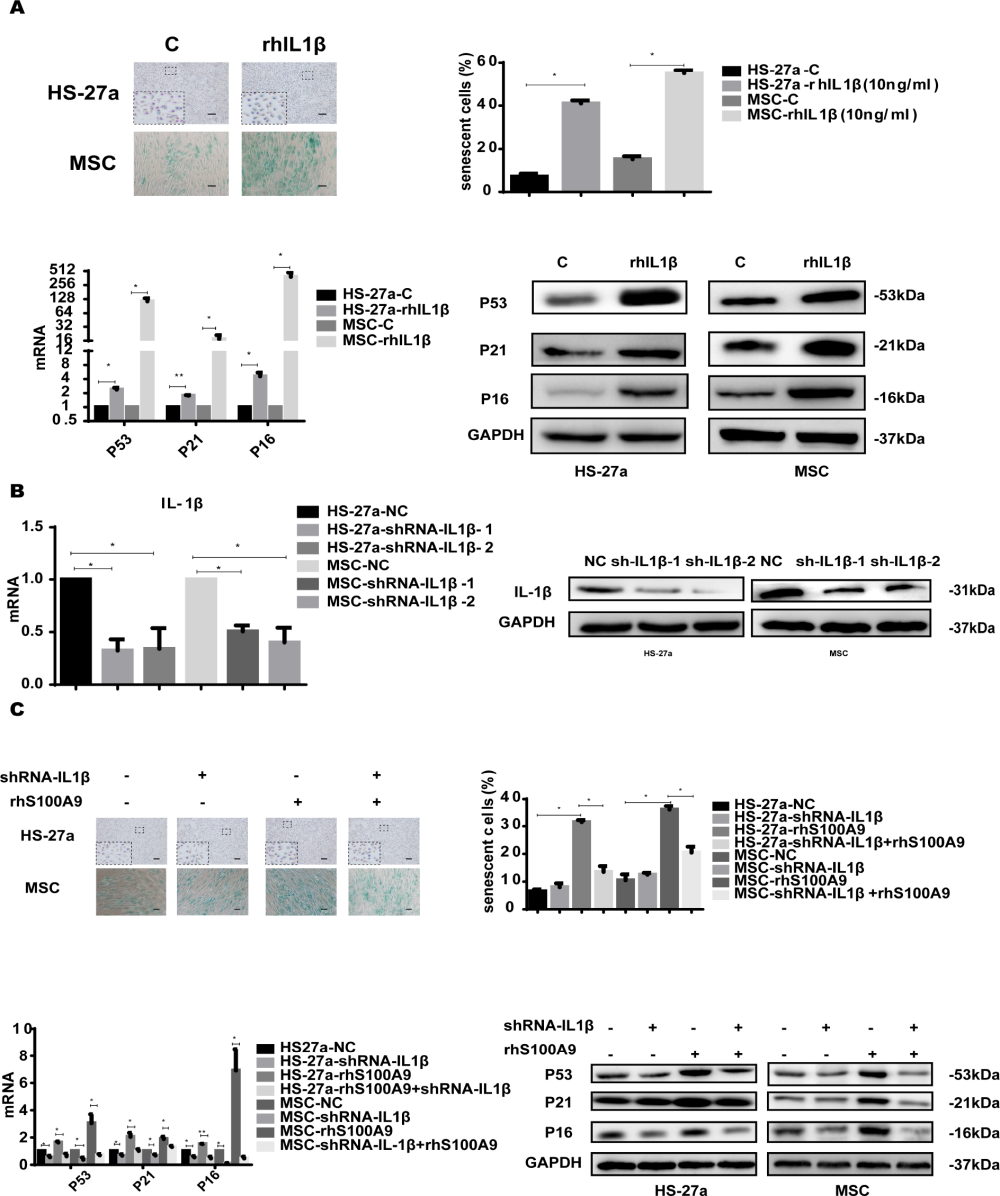
**IL-1β is sufficient to induce cellular senescence.** (**A**) Cells were treated with rhIL-1β (10 ng/ml) for 72h. Next, senescent cells were counted and presented in graphs (40 × magnification). The levels of p53, p21, and p16 were measured using qPCR and western blot. (**B**) RNAi efficiency of IL-1β was assayed utilizing qPCR and western blot. (**C**) IL-1β was knocked down, and cells were treated with S100A9 for 72h, senescent cells were counted and presented in graphs (40 × magnification). The expression of p53, p21 and p16 was determined using qPCR and western blot. Data are expressed as mean ± SD of three experiments. *P<0.05, **P<0.01, and ***P<0.001.

To investigate whether IL-1β mediated S100A9-induced cellular senescence in bone marrow stromal cells, we treated HS-27a and primary MSC cells with 10 ng/ml rhIL-1β. Subsequently, the proportion of senescent cells was significantly increased, and the expression of p53, p21, and p16 was upregulated as well (Figure 6A).

We further constructed two retrovirus-based RNAi vectors that transfected HS-27a and primary MSC cells with high efficiency, cells were infected with the retroviral supernatant containing shRNA specific to human IL-1β. On average, IL-1β was reduced by approximately 80% as evaluated by qPCR and western blot (Figure 6B). Moreover, IL-1β knockdown decreased the proportion of senescent cells and the p53, p21, and p16 levels induced by S100A9 (Figure 6C).

Taken together, our findings showed that MDS-derived S100A9 induces cellular senescence in mesenchymal stromal cells via the TLR4-NLRP3 inflammasome formation-IL-1β secretion signaling pathway.

## DISCUSSION

Tumors release numerous inflammatory mediators during their growth and infiltration [35]. Furthermore, various cytokines and transcription factors in the tumor inflammation microenvironment control the central tumor inflammatory signaling pathways and play a bridging role in tumor communication.

MDS is primarily an age-related neoplasm, and dysregulation of the innate immune signaling and inflammation is closely associated with the aging process [36]. Our previous studies have shown that MSCs in MDS, especially in lower-risk MDS, display a senescence phenotype [37, 38]. In this study, we revealed that S100A9 is highly-expressed in lower-risk MDS. Furthermore, HS-27a cells and normal primary MSCs co-cultured with lower-risk MDS-BM-MNC acquired senescence features, as evidenced by increased SA-β-X gal activity.

S100A9 secreted from tumor cells is an important regulator of the tumor microenvironment [12] and is an endogenous activator of TLR4. Our present study demonstrated that S100A9 induces cellular senescence in MSCs via TLR4.

Basiorka et al. revealed that S100A9 upregulates ROS production in hematopoietic cells and activates NLRP3 inflammasomes and β-catenin, resulting in ineffective hematopoiesis and MDS clone amplification. NLRs are a group of innate immunity molecules that sense extracellular and intracellular environmental changes and elicit inflammatory responses. Currently, the best-characterized inflammasome is the NLRP3 inflammasome. NLRP3 is a redox-sensitive cytosolic sensor that recruits the ASC adaptor protein and triggers ASC polymerization and nucleation of large cytoplasmic aggregates referred to as ASC specks, permitting docking and activation of pro–Caspase-1 that processes pro–IL-1β and pro–IL-18 to maturation and release [39–40].

The formation of the NLRP3 inflammasome is closely related to cellular senescence. In endothelial cells and cardiac fibroblasts, ROS production by various causes activates NLRP3 inflammasomes and further promotes cellular senescence [41–42]. NLRP3 inhibition in glioma cells reduces cellular senescence and suppresses tumor growth [43]. In the present study, we demonstrated that S100A9 promotes NLRP3 inflammasome formation and IL-1β secretion in HS-27a and primary MSC cells. Consistent with these in vitro results, We confirmed that NLRP3, Caspase-1, and IL-1β expression levels were also increased in lower-risk MDS.

ROS act as DAMP intermediates and are activated in MDS. In our study, S100A9 increased mitochondrial ROS level and reduced the anti-oxidant enzymes levels in HS-27a and primary MSC cells, and ROS depletion by DPI decreased NLRP3 inflammasome formation and IL-1β secretion. Furthermore, NLRP3 overexpression aggravated IL-1β secretion and cellular senescence.

IL-1β plays an important role in the interaction between tumor cells and their bone marrow microenvironment [44]. IL-1β produced by NLRP3 inflammasomes is a key molecule in cell senescence [45–48]. In our study, IL-1β induced cellular senescence in HS-27a and primary MSC cells, and IL-1β knockdown decreased cellular senescence induced by S100A9.

The disruption of inflammatory pathways has been associated with clonal selection and expansion in MDS [49], and immune dysregulation contributes to the pathogenesis of MDS [50]. The bone marrow microenvironment in patients with MDS appears to be inflammatory, a phenomenon mediated by the disruption of several pathways, which manifest the heterogeneity of the disease. Furthermore, early MDS samples demonstrated upregulated pro-inflammatory chemokine pathways [51]. Lower-risk MDS was correlated with more robust inflammatory cytokine profile characteristics and a greater degree of bone marrow apoptosis than higher-risk MDS. Contrarily, higher-risk MDS may be associated with downregulation of these cytokines, which may promote immune escape, vigorous clonal expansion, and an aggressive disease progress. Thus, we speculate that lack of inflammatory pathways in higher-risk MDS-MSCs may attenuate clonal clearance.

Our results demonstrated that MDS-derived S100A9 induces cellular senescence in MSCs through TLR4, NLRP3 inflammasome activation, and IL-1β secretion. Our findings deepen the understanding of the molecular mechanisms involved in MDS reprogramming of MSCs. Furthermore, our results indicate the essential role of S100A9 in tumor-environment interactions in bone marrow and greatly improve our understanding of the role of the NLRP3 inflammasome in cellular senescence. These insights may provide molecular targets for pharmacotherapeutic interventions in MDS

## MATERIALS AND METHODS

### Study participants

Patients were diagnosed as MDS in accordance with the minimum diagnostic criteria established by the conference on MDS [52]. 90 patients with MDS were included in this study; their characteristics are detailed in Supplementary Table 1. Patients were classified as “lower-risk” (IPSS-R<=3) and “higher-risk” (IPSS-R>=4.5) [53]. Furthermore, 30 healthy volunteers matched by gender and age were included as controls. All study participants provided written informed consent in accordance with the declaration of Helsinki. The study protocol was approved by the ethics committee of Shanghai Jiao Tong University affiliated Sixth People’s Hospital (SH6THHOSP- YS-2017-025).

### Isolation and culture of BM-MSCs

Bone marrow mononuclear cells (BM-MNCs) were isolated from fresh bone marrow aspirates and separated using Ficoll-Paque Plus (GE Healthcare, Uppsala, Sweden). BM-MNCs were seeded at a density of 1×10^6^ cells/ml and cultured in human mesenchymal stem cell growth medium (Cyagen Biosciences Inc, Guangzhou, China) supplemented with 10% fetal bovine serum (FBS), glutamine, and 100 U/ml penicillin/streptomycin at 37 °C with 5% CO_2_ in humidified atmosphere. After 72 h, the culture medium was replaced, and non-adherent cells were removed. Thereafter, the medium was exchanged twice per week. Upon achieving higher than 80% to 90% confluency, cells were detached using 0.25% trypsin–EDTA (Gibco, Grand Island, NY, USA). At the third passage (P3), adherent MSCs were harvested and utilized for experimental analyses.

### Cell lines and culture

The cell line HS-27a was maintained in RPMI-1640 medium supplemented with 10% FBS and 100 U/ml penicillin /streptomycin. Cells were maintained in humidified atmosphere containing 5% CO_2_ at 37 °C, and culture medium was replaced twice per week.

### Reagents

RhS100A9, CLI-095, rhIL-1β and DPI were purchased from Abcam (ab95909), Invivogen (CA92121), Pepro Tech (200-01B) and Selleck (S8639), respectively. Primary antibodies were obtained from the following manufacturers: p53, p21, and p16 from Cell Signalling Biotechnology (Danvers, MA, USA); NLRP3 from Abcam (Cambridge, MA, USA); and GAPDH, Caspase-1, and IL-1β from Affinity Biosciences. Alexa Fluor 594-conjugated goat anti-rabbit IgG (H + L) and FITC conjugated anti-rabbit IgG (H + L) secondary antibodies were purchased from Thermo Scientific (Rockford, IL, USA) and Affinity Biosciences, respectively. HRP conjugated secondary antibodies were purchased from Affinity Biosciences.

### Senescence-associated β-galactosidase (SA-β-gal) staining

Cells cultured on plates were washed with phosphate buffered saline (PBS) and fixed in 4% paraformaldehyde for 15 min at room temperature. After rinsing with PBS, cells were incubated with freshly prepared SA-β-Gal staining solution (Beyotime, China) for 16 h at 37 °C. Under a light microscope, the number of green (SA-β-Gal-positive) cells among at least 500 cells in 10 randomly chosen fields was counted to measure cellular senescence.

### Enzyme-linked immunosorbent assay (ELISA)

ELISA kit was purchased from Biotechnology Systems. Bone marrow supernatant and cell supernatant were collected and stored at −20 °C. ELISA was performed according to the manufacturer’s protocol.

### Real-time quantitative polymerase chain reaction (qPCR)

Total RNA was extracted using the RNeasy Mini Kit (Qiagen, Hilden, Germany), and cDNA was synthesized utilizing the Revert Aid TM First Strand cDNA Synthesis Kit (Fermentas, Burlington, Canada); both procedures were carried out according to the manufacturers’ protocols. qPCR was performed with Real Master Mix (Takara, Dalian, China) on an ABI 7500 real-time PCR machine (Applied Biosystems, Foster, CA, USA). Primer sequences used in the study are listed in Supplementary Table 2.

### Western blot

Cells were lysed on ice for 20 min in RIPA (Gibco, USA). Cell lysates were then centrifuged (12000 rpm/min), and supernatant were collected for western blot, equal amounts were separated with SDS-PAGE and blotted onto PVDF membranes. Membranes were incubated initially with primary antibodies and subsequently with corresponding secondary antibodies for 1 h. Specific bands were visualized using enhanced chemiluminescence (ECL) western blotting detection kit (Amersham Biosciences).

### Virus transfection assay

ShRNA-TLR4, shRNA-IL-1β, and NLRP3-OE lentiviruses were purchased from Genechem Company (Shanghai, China). Transfection was performed according to the manufacturer’s protocol.

### Immunofluorescence assay

Cells were seeded onto 20 mm round coverslips and fixed at 37 °C for 10 min using 4% paraformaldehyde, and washed with PBS. Next, cells were permeabilized with 0.1% Triton X-100 in PBS for 15 min at room temperature, washed with PBS, blocked with 2% BSA in PBS for 30 min at room temperature, and washed again. Cells were incubated with the appropriate primary antibody overnight (dilution 1:200 for NLRP3 and 1:400 for Caspase-1) at 4 °C. On the following day, cells were washed with PBS and incubated with the appropriate secondary antibodies (1:500) for 1 h at room temperature. After washing, cells were stained with DAPI. Images were captured under a confocal microscope (Leica TCS SP8 STED) and analysed with Image-Pro Plus 6.0.

### Statistical analyses

All experiments were repeated at least three times. The results of multiple experiments were presented as mean ± standard deviation (SD). Statistical analyses were performed using GraphPad Prism version 6.0. P-values were calculated utilizing Student’s t-test or one-way analysis of variance (ANOVA). P-value <0.05 was considered statistically significant.

## Supplementary Material

Supplementary Methods

Supplementary Figure 1

Supplementary Table 1

Supplementary Table 2
