# Supplementary material for: Cellular senescence induced by S100A9 in mesenchymal stromal cells through NLRP3 inflammasome activation
Source: Aging (Albany NY). 2019 Nov 14;11(21):9626–42. doi: 10.18632/aging.102409 (PMC6874461; doi:10.18632/aging.102409)
Supplement: Supplementary Methods [file aging-11-102409-s003.pdf]

SUPPLEMENTARY METHODS

Mitochondrial ROS assay

Cells, seeded in 6-hole plate, were incubated with 1.0 ml of 5 μM solution of MitoSOXred (ThermoFisher Scientific, USA) for 10 min at 37 °C in the dark and washed with PBS. Then, stained with DAPI and analysed using a fluorescence microscope.

Analysis of antioxidant substances

Cells were homogenized by ultrasonic vibration and centrifuged for 12000 rpm/min, 10 min, the supernatant was collected, and the levels of superoxide dismutase (SOD), catalase (CAT), and glutathione (GSH) were measured according to the manufacturer's instructions (Jiancheng biotech, Nanjing, Jiangsu, China).
