# Supplementary material for: Cellular senescence induced by S100A9 in mesenchymal stromal cells through NLRP3 inflammasome activation
Source: Aging (Albany NY). 2019 Nov 14;11(21):9626–42. doi: 10.18632/aging.102409 (PMC6874461; doi:10.18632/aging.102409)
Supplement: Supplementary Figure 1 [file aging-11-102409-s004.pdf]

SUPPLEMENTARY FIGURE

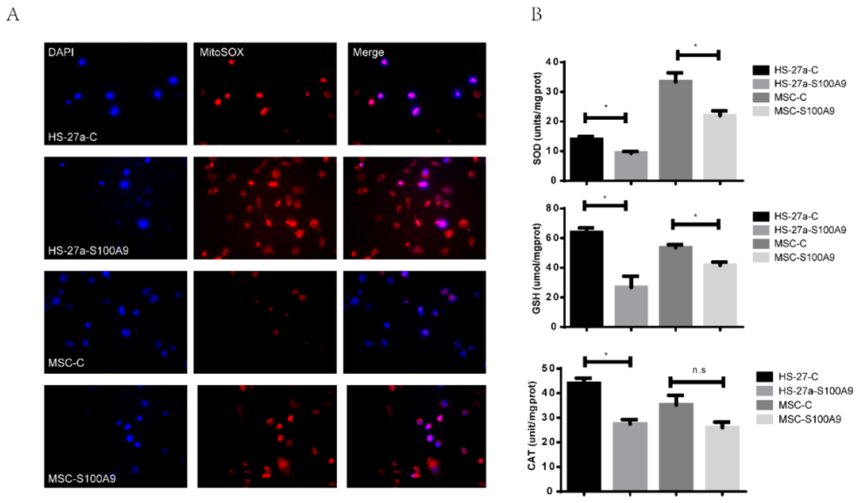

Supplementary Figure 1. Increased mitochondrial ROS and reduced anti-oxidant enzymes levels in S100A9-induced HS-27a and primary MSC cells. cells were treated with rhS100A9 for 72h, mitochondrial ROS (**A**) was measured with fluorescence microscope (DAPI, blue; MitoSOX, red); and superoxide dismutase (SOD), glutathione (GSH) , and catalase (CAT) levels (**B**) were tested.
