# Supplementary material for: Cellular senescence induced by S100A9 in mesenchymal stromal cells through NLRP3 inflammasome activation
Source: Aging (Albany NY). 2019 Nov 14;11(21):9626–42. doi: 10.18632/aging.102409 (PMC6874461; doi:10.18632/aging.102409)
Supplement: Supplementary Table 1 [file aging-11-102409-s001.docx]

**Supplementary Table 1. The characteristics of patients who were enrolled in the study.**

| Parameter | | |
| --- | --- | --- |
| Sex (median value) | Male | 57 |
|  | Female | 55 |
| Median age, years (range) | 56 (14–82) | |
| WHO classifcation | RCUD | 12 |
|  | RARS | 10 |
|  | RCMD | 17 |
|  | RAEB-1 | 19 |
|  | RAEB-2 | 11 |
|  | MDS-U | 3 |
|  | 5q- MDS | 4 |
| IPSS | <=0.5 | 18 |
|  | >=1.5 | 12 |
| IPSS-R | <=3 | 25 |
|  | >=4.5 | 21 |

**BM-MNC (bone marrow mononuclear cells)**

| NO. | DIAGNOSIS | AGE | SEX | IPSS-R | KARYOTYPE |
| --- | --- | --- | --- | --- | --- |
| 1 | Anemia | 35 | Female | / | Normal |
| 2 | Anemia | 56 | Female | / | Normal |
| 3 | Anemia | 43 | Male | / | Normal |
| 4 | Anemia | 31 | Female | / | Normal |
| 5 | ITP | 52 | Male | / | Normal |
| 6 | ITP | 23 | Female | / | Normal |
| 7 | Anemia | 65 | Female | / | Normal |
| 8 | Anemia | 43 | Female | / | Normal |
| 9 | Anemia | 21 | Male | / | Normal |
| 10 | ITP | 47 | Female | / | Normal |
| 11 | Anemia | 57 | Female | / | Normal |
| 12 | Anemia | 63 | Male | / | Normal |
| 13 | Anemia | 52 | Female | / | Normal |
| 14 | Anemia | 54 | Male | / | Normal |
| 15 | ITP | 44 | Female | / | Normal |
| 16 | ITP | 52 | Male | / | Normal |
| 17 | ITP | 43 | Female | / | Normal |
| 18 | Anemia | 75 | Female | / | Normal |

| NO. | DIAGNOSIS | AGE | SEX | IPSS-R | KARYOTYPE |
| --- | --- | --- | --- | --- | --- |
| 1 | LR-MDS | 63 | Male | 2 | Normal |
| 2 | LR-MDS | 39 | Female | 3 | Normal |
| 3 | LR-MDS | 14 | Female | 1.5 | Normal |
| 4 | LR-MDS | 35 | Female | 3 | Normal |
| 5 | LR-MDS | 67 | Male | 2 | Normal |
| 6 | LR-MDS | 57 | Female | 3 | Normal |
| 7 | LR-MDS | 34 | Female | 3 | Normal |
| 8 | LR-MDS | 53 | Female | 2.5 | Normal |
| 9 | LR-MDS | 62 | Female | 3 | Normal |
| 10 | LR-MDS | 59 | Male | 2.5 | Normal |
| 11 | LR-MDS | 57 | Male | 3 | Normal |
| 12 | LR-MDS | 41 | Male | 2.5 | Normal |
| 13 | LR-MDS | 69 | Male | 3 | Normal |
| 14 | LR-MDS | 43 | Male | 2.5 | Normal |
| 15 | LR-MDS | 65 | Female | 3 | Normal |
| 16 | LR-MDS | 68 | Male | 1.5 | Normal |
| 17 | LR-MDS | 48 | Female | 2 | Normal |
| 18 | LR-MDS | 61 | Male | 3 | Normal |
| 19 | LR-MDS | 56 | Male | 3 | Normal |
| 20 | LR-MDS | 46 | Female | 2.5 | Normal |
| 21 | LR-MDS | 67 | Male | 1 | Normal |
| 22 | LR-MDS | 58 | Female | 1.5 | Normal |
| 23 | LR-MDS | 59 | Female | 2 | Normal |
| 24 | LR-MDS | 48 | Female | 3 | Normal |
| 25 | LR-MDS | 55 | Male | 3 | Normal |

| NO. | DIAGNOSIS | AGE | SEX | IPSS-R | KARYOTYPE |
| --- | --- | --- | --- | --- | --- |
| 1 | HR-MDS | 43 | Female | 4.5 | 46,XX,del(5)(q31)[6]/46,XX[10] |
| 2 | HR-MDS | 56 | Male | 5 | Normal |
| 3 | HR-MDS | 76 | Male | 4.5 | Normal |
| 4 | HR-MDS | 67 | Female | 4.5 | Normal |
| 5 | HR-MDS | 66 | Female | 6.5 | Normal |
| 6 | HR-MDS | 78 | Male | 6.5 | Normal |
| 7 | HR-MDS | 63 | Male | 4.5 | Normal |
| 8 | HR-MDS | 52 | Male | 7 | 47,xy,+8[3]/46,xy[17] |
| 9 | HR-MDS | 58 | Female | 5 | 46,xy,inv(1)(q21q25),del(5)(q22q35)[9]/46,xy[9] |
| 10 | HR-MDS | 78 | Female | 4.5 | 46,xx,-1,add(3)(q13.2),add(5)(q11.2),?add(7)(q22),-9,+11,add(11)(p11.2),-17,-20,+mar1,+mar2,+mar3[2]/46,xx[3] |
| 11 | HR-MDS | 62 | Female | 5.5 | Normal |
| 12 | HR-MDS | 55 | Male | 4.5 | 43,x,-y,-5,-13,-17,-21,+mar1,+mar2[7]/43,x,-y,add(5)(q11.2),-7,-17,-19,-21,+mar1,+mar3[4]/46,xy[6] |
| 13 | HR-MDS | 41 | Female | 6 | Normal |
| 14 | HR-MDS | 56 | Male | 5.5 | 46,xy,del(5)(q15q35)[5]/46,idem,der(18;21)(q10;q10),+21[14] |
| 15 | HR-MDS | 63 | Male | 8 | 46,xy,?add(3),add(5)(q11.2)[5]/46,xy[2] |
| 16 | HR-MDS | 63 | Male | 5.5 | 46,,xy,del(20)(q11.2q13.3)[20] |
| 17 | HR-MDS | 59 | Male | 4.5 | 44,xy,del(2)(p21),add(5)(q13),der(7;17)(p10,q10),-9,?add(9)(p24),add(12)(p11.2),-20,+mar[14]/46,xy[6] |
| 18 | HR-MDS | 76 | Male | 7 | Normal |
| 19 | HR-MDS | 69 | Female | 4.5 | Normal |
| 20 | HR-MDS | 56 | Female | 4.5 | 48,xx,add(1)(q21),-3,del(5)(q13q33),-7,+11,-12,-18,+mar1,+mar2,+mar3,+mar4,+mar5[4]/46,xx,add(1)(q21),-3,del(5)(q13q33),del(6)(q10),-7,+11,-12,-18,+mar1,+mar2,+mar6[2]/46,xx[14] |
| 21 | HR-MDS | 76 | Male | 4.5 | 46,XY,dup(1)(q21q32),?del(7q)[3] |

**MSC (mesenchymal stromal cells)**

| NO. | DIAGNOSIS | AGE | SEX | IPSS | KARYOTYPE |
| --- | --- | --- | --- | --- | --- |
| 1 | ITP | 19 | Female | / | Normal |
| 2 | ITP | 35 | Female | / | Normal |
| 3 | Normal | 31 | Male | / | Normal |
| 4 | Normal | 28 | Male | / | Normal |
| 5 | Normal | 50 | Female | / | Normal |
| 6 | Normal | 78 | Female | / | Normal |
| 7 | Normal | 63 | Female | / | Normal |
| 8 | Normal | 35 | Female | / | Normal |
| 9 | Normal | 46 | Male | / | Normal |
| 10 | Normal | 42 | Female | / | Normal |
| 11 | Anemia | 72 | Male | / | Normal |
| 12 | Anemia | 45 | Female | / | Normal |
| 13 | Drug-induced Myelosuppression | 82 | Male | / | Normal |
| 14 | Normal | 57 | Male | / | Normal |
| 15 | Normal | 52 | Male | / | Normal |
| 16 | Normal | 24 | Female | / | Normal |
| 17 | Normal | 26 | Male | / | Normal |
| 18 | Normal | 24 | Male | / | Normal |

| NO. | DIAGNOSIS | AGE | SEX | IPSS | KARYOTYPE |
| --- | --- | --- | --- | --- | --- |
| 1 | LR-MDS | 42 | Female | 0.5 | Normal |
| 2 | LR-MDS | 61 | Female | 0 | 47,xx,+8[20] |
| 3 | LR-MDS | 67 | Female | 0 | Normal |
| 4 | LR-MDS | 68 | Male | 0.5 | Normal |
| 5 | LR-MDS | 30 | Male | 0 | Normal |
| 6 | LR-MDS | 77 | Male | 0 | Normal |
| 7 | LR-MDS | 64 | Female | 0 | 47，xx,+8[15]/46，xx[7] |
| 8 | LR-MDS | 62 | Male | 0 | Normal |
| 9 | LR-MDS | 36 | Female | 0.5 | Normal |
| 10 | LR-MDS | 68 | Male | 0.5 | Normal |
| 11 | LR-MDS | 21 | Female | 0.5 | Normal |
| 12 | LR-MDS | 46 | Male | 0 | Normal |
| 13 | LR-MDS | 46 | Female | 0.5 | Normal |
| 14 | LR-MDS | 25 | Male | 0.5 | Normal |
| 15 | LR-MDS | 62 | Female | 0 | Normal |
| 16 | LR-MDS | 61 | Female | 0.5 | Normal |
| 17 | LR-MDS | 63 | Female | 0.5 | Normal |
| 18 | LR-MDS | 55 | Male | 0.5 | Normal |

| NO. | DIAGNOSIS | AGE | SEX | IPSS | KARYOTYPE |
| --- | --- | --- | --- | --- | --- |
| 1 | HR-MDS | 56 | Male | 1.5 | 46,xy[qh-)[10] |
| 2 | HR-MDS | 65 | Male | 2 | 45,x,-y,inv(3)(q21q26)t(4;11)(q21;23)[8] |
| 3 | HR-MDS | 73 | Male | 2.5 | Normal |
| 4 | HR-MDS | 30 | Male | 1.5 | Normal |
| 5 | HR-MDS | 48 | Male | 1.5 | Normal |
| 6 | HR-MDS | 49 | Male | 1.5 | Normal |
| 7 | HR-MDS | 48 | Male | 1.5 | Normal |
| 8 | HR-MDS | 57 | Male | 2 | Normal |
| 9 | HR-MDS | 72 | Female | 2 | Normal |
| 10 | HR-MDS | 36 | Female | 1.5 | Complex karyotype |
| 11 | HR-MDS | 52 | Male | 1.5 | Normal |
| 12 | HR-MDS | 67 | Male | 1.5 | Normal |
