# Supplementary material for: Cellular senescence induced by S100A9 in mesenchymal stromal cells through NLRP3 inflammasome activation
Source: Aging (Albany NY). 2019 Nov 14;11(21):9626–42. doi: 10.18632/aging.102409 (PMC6874461; doi:10.18632/aging.102409)
Supplement: Supplementary Table 2 [file aging-11-102409-s002.pdf]

Supplementary Table 2. Primer sets for qPCR.

Name	Primer Sequence (5’ to 3’)
NLRP3-F	CAATGGGGAGGAGAAGGCGT
NLRP3-R	TCTGAACCCCACTTCGGCTC
Caspase1-F	TGAGCAGCCAGATGGTAGAGC
Caspase1-R	TCACTTCCTGCCCACAGACAT
IL-1β-F	CTCTTCGAGGCACAAGGCAC
IL-1β-R	CAAGTCATCCTCATTGCCACTGT
IL-6-F	ACTCACCTCTTCAGAACGAATTG
IL-6-R	CCATCTTTGGAAGGTTCAGGTTG
IL-8-F	TTTTGCCAAGGAGTGCTAAAGA
IL-8-R	AACCCTCTGCACCCAGTTTTC
TGFβ-F	GGCCAGATCCTGTCCAAGC
TGFβ-R	GTGGGTTTCCACCATTAGCAC
P53-F	CAGCACATGACGGAGGTTGT
P53-R	TCATCCAAATACTCCACACGC
P21-F	TGTCCGTCAGAACCCATGC
P21-R	AAAGTCGAAGTTCCATCGCTC
P16-F	GATCCAGGTGGGTAGAAGGTC
P16-R	CCCCTGCAAACTTCGTCCT
S100A9-F	TGGCTCCTCGGCTTTGACAGAGT
S100A9-R	TGGGTGCCCCAGCTTCACAGA
TLR4-F	TGCGTGAGACCAGAAAGC
TLR4-R	TTAAAGCTCAGGTCCAGGTTC
GAPDH-F	GCACCGTCAAGGCTGAGAAC
GAPDH-R	GTGGTGAAGACGCCAGTGGA
